# The hidden role of left atrial strain: insights into end-organ damage in dipper and nondipper hypertension

**DOI:** 10.1038/s41371-025-01017-5

**Published:** 2025-04-12

**Authors:** Özden Seçkin, Serkan Ünlü, Mehmet Rıdvan Yalçın

**Affiliations:** Gazi University Hospital Cardiology Department, Emniyet, Mevlana Blv. No:29, 06560 Yenimahalle Ankara, Turkey

**Keywords:** Hypertension, Kidney diseases

## Abstract

Hypertension is a major global health concern associated with significant morbidity and mortality due to its effects on target organs such as the heart, kidneys, and brain. Non-dipper hypertension, characterized by insufficient nocturnal blood pressure reduction leading to sustained hemodynamic load, is linked to higher risks of end-organ damage. This study evaluates the impact of hypertension on LA anatomy and physiomechanics and explores the association of LA strain parameters with hypertensive heart disease and nephropathy. This prospective, single-center cohort study included 135 participants: 35 healthy controls, 50 dipper hypertension, and 50 non-dipper hypertension patients. All participants underwent 24-h ABPM, biochemical analysis, and transthoracic echocardiography, including speckle-tracking echocardiography, a novel technique for LA strain analysis. LA diameter,LASr, and LAVI maximum were identified as significant parameters associated with hypertensive end-organ damage. Non-dipper hypertension was associated with significantly higher 24-h blood pressure values and reduced nocturnal dipping compared to dipper hypertension. LA strain parameters(LASr, LAScd, LASct) were significantly lower in the non-dipper group. These reductions indicate impaired left atrial mechanics and early cardiac dysfunction. Logistic regression revealed that LA diameter, LASr, and LAVI maximum were significant variables associated with hypertensive heart disease, whereas only LASr was significantly associated with hypertensive nephropathy. LA strain analysis provides valuable insights into the pathophysiology of hypertension and its complications. The findings support the use of LA parameters as cost-effective, noninvasive biomarkers for assessing associations with hypertensive heart disease and nephropathy, enabling early risk stratification. This emphasizes the importance of enhanced monitoring and tailored interventions for high-risk patients.

## Introduction

Hypertension is a leading global health challenge, significantly contributing to cardiovascular morbidity and mortality [1]. Among its subtypes, non-dipper hypertension has emerged as a critical risk factor for end-organ damage, demanding more detailed investigation [2, 3]. Previous studies have established the superiority of 24-h ambulatory blood pressure monitoring (ABPM) over office blood pressure measurements in both diagnostic and prognostic evaluations [4]. ABPM offers a unique ability to assess circadian blood pressure variability, providing superior insights into nocturnal dipping patterns [5].

Physiologically, during nocturnal rest, increased vagal tone and decreased sympathetic activity lead to significant reductions in heart rate, cardiac output, and peripheral vascular resistance, resulting in over 10% blood pressure reduction [6]. When this nocturnal blood pressure drop is less than 10%, the condition is defined as “non-dipper hypertension” [7]. Numerous studies have linked non-dipper hypertension to a higher prevalence of end-organ damage, including atherosclerotic vascular disease, left ventricular hypertrophy, heart failure, proteinuria, kidney failure, and stroke [4, 8–10].

Systemic hypertension affects the left atrium through hemodynamic mechanisms, such as elevated left ventricular filling pressures, and neurohormonal pathways involving increased RAAS and BNP levels [11, 12]. This contributes to left atrial remodeling, with left atrial enlargement as the earliest detectable change in conventional echocardiography. However, volumetric assessment is insufficient to capture early impairments in left atrial physiomechanics [13, 14]. The physiological roles of the left atrium encompass functioning as a reservoir during ventricular systole, a conduit during early diastole and diastasis, and a pump during late diastole [15, 16].

In the past decade, speckle-tracking echocardiography has emerged as a reliable and reproducible technique for noninvasively assessing atrial physiomechanics with angle-independent measurements that are less affected by loading conditions [17, 18]. This study aims to provide a comprehensive evaluation of end-organ damage and to investigate the association of left atrial phasic functions with end-organ damage.

## Materials and methods

### Study design and ethics

This prospective, single-center cohort study was conducted at Gazi University Hospital in accordance with the principles of the Helsinki Declaration. Ethical approval was obtained from the Gazi University Non-Interventional Clinical Research Ethics Committee (Approval No:401), and written informed consent was collected from all participants.

### Study population

Between March 2021 and May 2024, a total of 135 participants were recruited, including 35 healthy controls, 50 dipper hypertensive, and 50 non-dipper hypertensive patients. Participants were aged 18 years or older, newly diagnosed with hypertension, and not receiving any antihypertensive treatments. Exclusion criteria included cardiomyopathy, coronary artery disease, significant valvular heart disease (except mild mitral regurgitation), diabetes mellitus, renal disease, and pregnancy. These inclusion and exclusion criteria were pre-specified before patient enrollment to ensure a homogeneous study population.

### Echocardiographic assessment and measurements

Conventional 2D echocardiography was performed using a General Electrics Vivid E95 system with an M5Sc-D transducer (GE Vingmed Ultrasound, Norway), following the American Society of Echocardiography guidelines [19]. Left atrial volumes were measured as LAVmax at end-systole and LAVmin at late diastole. Left ventricular mass index (LVMI) was calculated using the formula: 0.8 × (1.04 × [(IVS + LVID + PWT)^3^ − LVID^3^] + 0.6) and indexed to body surface area.

For global longitudinal strain (LV-GLS) analysis, apical four- and two-chamber images were acquired during expiration and analyzed using EchoPac v201 software. The reference point was placed at the onset of the QRS complex, with regional tracking manually validated.

### Left atrial strain measurements

**Fig. 1:**
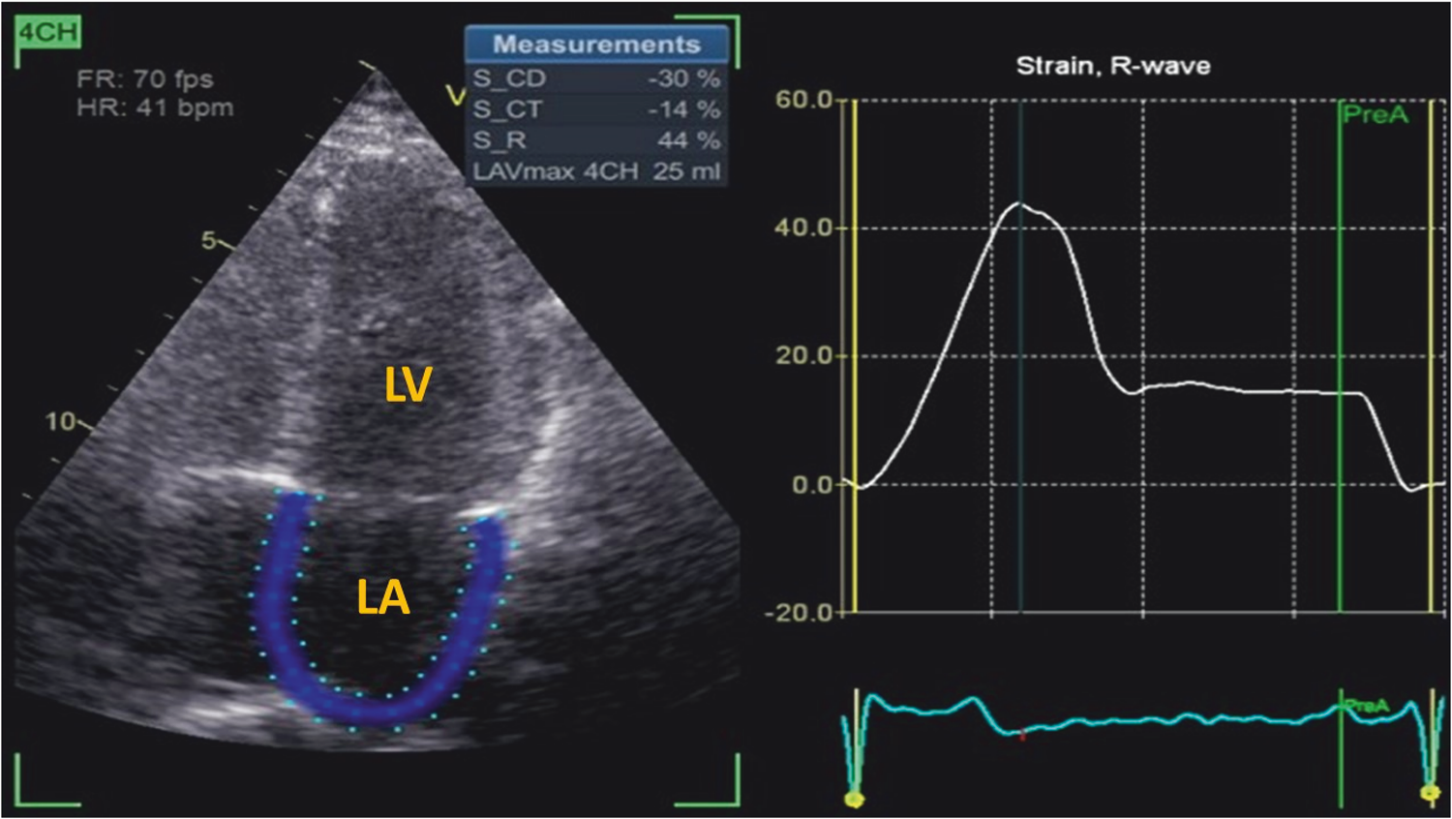
Left atrial strain analysis using two-dimensional speckle tracking echocardiography in a control group patient. LA left atrium, LV left ventricle. The region of interest (ROI) in the left atrium is marked in thick blue. The adjacent panel shows the left atrial strain curve, with a white line indicating global strain. Representative strain parameters are S_CD (conduit phase): 30%, S_CT (contractile phase): -14%, and S_R (reservoir phase): 44%.

Left atrial strain analysis was performed using 2D speckle-tracking echocardiography (2D STE) to evaluate left atrial phasic functions comprehensively. Apical two- and four-chamber views were acquired using a frame rate of 50–80 frames per second during quiet respiration. A semi-automated software (EchoPac v201) was used to delineate the endocardial borders of the left atrium, ensuring exclusion of the pericardium from the region of interest (ROI). The ROI was adjusted manually if necessary to maintain accurate tracking (Fig. 1).

For strain measurements, the cardiac cycle was defined from the onset of the QRS complex to the subsequent QRS complex, using R-R gating, capturing the full phasic cycle of the left atrium. Strain measurements were calculated for the following phases: reservoir strain (LASr), conduit strain (LAScd), and contraction strain (LASct). Strain parameters were expressed as percent changes relative to the baseline dimension and reported as the mean of three consecutive cardiac cycles to reduce variability. The tracking quality was validated visually, and segments with poor tracking were excluded. All analyses were conducted by a single investigator blinded to the patient groups to ensure consistency and reduce bias.

### Definition of end-organ damage in hypertension

Microalbuminuria was defined as a morning spot urine albumin-to-creatinine ratio of 30–300 mg/g, as recommended by the guidelines, classified as microalbuminuria. Values below 30 mg/g were classified as normoalbuminuria [20, 21]. The morning spot urine method is used for its simplicity and wide acceptance in clinical practice, despite some limitations in capturing daily variations [22].

Hypertensive heart disease (HHD) in this study was defined as the presence of left ventricular hypertrophy (LVH) and/or left atrial enlargement (LAE) identified through echocardiographic parameters, in conjunction with clinical evidence of systemic hypertension. Specifically, LVH was diagnosed based on a left ventricular mass index (LVMI) exceeding 115 g/m^2^ in men or 95 g/m^2^ in women, while LAE was determined using a left atrial volume index (LAVI) > 34 mL/m^2^ [23, 24]. These criteria reflect the structural adaptations and remodeling caused by chronic pressure overload and hemodynamic stress in hypertensive patients.

### Statiscal analysis

Numerical variables were summarized as mean ± standard deviation (SD) for normally distributed data or as median (interquartile range, IQR) for data that were not normally distributed. Normality of distribution was assessed using the Kolmogorov–Smirnov test. Categorical variables were presented as numbers and percentages.

Intergroup comparisons of numerical variables were performed using one-way analysis of variance (ANOVA) for normally distributed variables. Post hoc Bonferroni tests were applied to assess specific group differences where ANOVA indicated statistical significance. For non-normally distributed variables, the Kruskal-Wallis test was used, followed by pairwise comparisons with adjusted *p*-values. Categorical variables were compared using the chi-square test.

To assess the association between clinical and echocardiographic parameters with the presence of hypertensive heart disease or nephropathy, univariate and multivariate logistic regression analyses were conducted. Variables showing significant correlations in univariate analysis (*p* < 0.1) and clinically relevant covariates, such as age and sex, were entered into multivariate logistic regression models. Stepwise backward elimination was utilized to refine the models, retaining variables with a *p*-value < 0.05.

All *p*-values were two-tailed, and statistical significance was defined as *p* < 0.05. Data analysis was performed using SPSS software (version 23.0; IBM Corp., Armonk, NY, USA).

The sample size was determined using a power analysis to detect a pre-specified effect size with α = 0.05 and a power of 80%. The estimated effect size was derived from previous studies evaluating left atrial strain in hypertensive patients [25, 26].

## Results

### Demographic data and conventional-tissue doppler echocardiographic findings

The study included 135 participants: 35 healthy controls, 50 dipper hypertensive patients, and 50 non-dipper hypertensive patients. The groups were comparable in terms of age, sex, smoking status, body mass index (BMI), and body surface area (BSA) (*p* > 0.05). However, significant differences were observed in blood pressure parameters. Daytime, nighttime, and 24-h average systolic and diastolic blood pressures were significantly higher in the dipper and non-dipper hypertension groups compared to the control group (*p* < 0.001). In the non-dipper hypertension group, the reduction in nighttime systolic and diastolic blood pressure was markedly lower (*p* < 0.001 for both, Table 1).

**Table 1:** Baseline Demographic and Clinical Characteristics of Study Groups.

Variables	Control group (n = 35)	Dipper HT group (n = 50)	Non-dipper HT group (n = 50)	*p*
Age	56.1 ± 6.8	56.3 ± 8.3	56.7 ± 6.3	0.912
Female (n, (%))	16 (45.7)	22 (44)	24 (48)	0.922
Smoking (n, (%))	12 (34.2)	21 (42)	23 (46)	0.556
BMI (kg/m^2^)	28.7 ± 2	29 ± 2.4	29 ± 2.4	0.816
BSA (m^2^)	1.78 ± 0.11	1.79 ± 0.1	1.77 ± 0.12	0.408
Daytime Average Systolic BP	117 ± 9.3^a,c^	165.2 ± 11.4^b,c^	164 ± 12.9^a,b^	<0.001
Nighttime Average Systolic BP	101.8 ± 10.4^a,c^	137.7 ± 10.6^b,c^	153.3 ± 12.5^a,b^	<0.001
Daytime Average Diastolic BP	73.9 ± 7.8^a,c^	97.1 ± 9.9^b,c^	94.4 ± 5.1^a,b^	<0.001
Nighttime Average Diastolic BP	58.8 ± 6.4^a,c^	83.1 ± 10^b,c^	90.4 ± 5.1^a,b^	<0.001
24-h Average Systolic BP	113.1 ± 9.5^a,c^	157.4 ± 11.1^b,c^	161.3 ± 12.7^a,b^	<0.001
24-h Average Diastolic BP	113.1 ± 9.5^a,c^	97.1 ± 8.3^b,c^	88.1 ± 9.6^a,b^	<0.001
Nocturnal Dip Systolic	13.6 ± 4.2^a,c^	16.6 ± 2.2^a,c^	6.5 ± 2.0^a,b^	<0.001
Nocturnal Dip Diastolic	20.1 ± 6.7^a,c^	14.5 ± 1.5^b,c^	4.2 ± 0.2^a,b^	<0.001
Hypertensive heart disease	0 (0)^a,c^	28 (56)^b,c^	36 (72)^a,b^	<0.001
Hypertensive nephropathy	0 (0)^a,c^	11 (22)^b,c^	27 (54)^a,b^	<0.001

*HT* Hypertension, *BMI* body mass index, *BSA* body surface area, *BP* blood pressure, *BP* blood pressure.

ANOVA test was used for continuous variables with post-Hoc Bonferroni correction. Categorical variables were compared by the x2 test. For post-hoc analysis results are shown as;

^a^Significantly different compared to the Dipper HT group.

^b^Significantly different compared to the Control group.

^c^Significantly different compared to the Non-dipper HT group.

Biochemical analysis showed significantly higher microalbuminuria levels in the non-dipper hypertension group (89.1 ± 18.5 mg/g) compared to the dipper hypertension (48.5 ± 14.5 mg/g) and control groups (19.3 ± 4.6 mg/g, *p* < 0.001). Fasting blood glucose levels also differed significantly, with the control group showing higher values compared to the dipper hypertension group (*p* = 0.034). Other parameters, such as lipid profiles and creatinine levels, were comparable across groups (p > 0.05, Table 2).

**Table 2:** Biochemical and Hematological Parameters of Study Groups.

Variables	Control group (n = 35)	Dipper HT group (n = 50)	Non-dipper HT group (n = 50)	*p*
Total Cholesterol (mg/dL)	177 ± 40.1	179 ± 31.7	182.9 ± 38.1	0.744
LDL Cholesterol (mg/dL)	101.2 ± 35	99.2 ± 28.7	101.9 ± 30.3	0.905
HDL Cholesterol (mg/dL)	50.2 ± 12.9	49.3 ± 13.9	49.4 ± 10.8	0.944
Triglycerides (mg/dL)	155.5 ± 100.8	152.6 ± 91.8	158.2 ± 72.2	0.951
Creatinine (mg/dL)	0.81 ± 0.25	0.88 ± 0.32	0.91 ± 0.38	0.438
Fasting Blood Glucose (mg/dL)	96.4 ± 15.2^a,c^	89.4 ± 10.6^b,c^	91.2 ± 11.3^a,b^	0.034
Microalbuminuria (mg/g creatinine)	19.3 ± 4.6^a,c^	48.5 ± 14.5^b,c^	89.1 ± 18.5^a,b^	<0.001

*HT* hypertension, *LDL* low-density lipoprotein, *HDL* high-density lipoprotein, *BP* blood pressure, Microalbuminuria: Urinary Albumin-to-Creatinine Ratio.

ANOVA test was used for continuous variables with post-Hoc Bonferroni correction. Categorical variables were compared by the x2 test. For post-hoc analysis results are shown as;

^a^Significantly different compared to the Dipper HT group.

^b^Significantly different compared to the Control group.

^c^Significantly different compared to the Non-dipper HT group.

The prevalence of hypertensive heart disease was 72% in the non-dipper hypertension group, 56% in the dipper hypertension group, and 0% in the control group (*p* < 0.001, Table 1). Similarly, the rate of hypertensive nephropathy was 54% in the non-dipper group, 22% in the dipper group, and 0% in the control group (*p* < 0.001, Table 1).

### Left ventricular global longitudinal strain (LV-GLS) analysis

There were no statistically significant differences in left ventricular global longitudinal strain (LV-GLS) values among the groups (*p* = 0.324). LV-GLS values showed a similar distribution across all groups (Table 3).

**Table 3:** Echocardiographic Measurements of Study Groups.

Variables	Control group (n = 35)	Dipper HT group (n = 50)	Non-dipper HT group (n = 50)	*p*
LA diameter (mm)	32.3 ± 3.7^a,c^	38.9 ± 2.5^b,c^	41.1 ± 2.8^a,b^	<0.001
LAVI minimum (mL/m^2^)	4.5 ± 1.0^a,c^	5.2 ± 1.9^b,c^	7.2 ± 2.0^a,b^	<0.001
LAVI maximum (mL/m^2^)	10.4 ± 1.8^a,c^	11.4 ± 1.7^b,c^	12.4 ± 1.6^a,b^	<0.001
IVS thickness (mm)	9.7 ± 0.5^a,c^	11.2 ± 0.8^b,c^	13.5 ± 1.2^a,b^	<0.001
PW thickness (mm)	9.1 ± 0.5^a,c^	10.5 ± 0.8^b,c^	12.2 ± 1.1^a,b^	<0.001
LVED diameter (mm)	42.8 ± 2.8	43.9 ± 3.3	44.2 ± 4.0	0.169
EF (%)	63.8 ± 2.7	63.9 ± 3.2	64.4 ± 2.6	0.591
LVMI (g/m^2^)	99.0 ± 16^a,c^	132.7 ± 27^b,c^	135.9 ± 28	<0.001
LVGLS (%)	17.2 ± 1.76	16.6 ± 2	16.7 ± 2.01	0.324

*HT* hypertension, *LA* left atrium, *LAVI* left atrial volume index, *IVS* Interventricular Septum, *PW* posterior wall, *LVED* left ventricular end-diastolic, *EF* ejection fraction, *LVMI* left ventricular mass index, *LVGLS* left ventricular global longitudinal strain.

ANOVA test was used for continuous variables with post-Hoc Bonferroni correction. Categorical variables were compared by the x2 test. For post-hoc analysis results are shown as;

^a^Significantly different compared to the Dipper HT group.

^b^Significantly different compared to the Control group.

^c^Significantly different compared to the Non-dipper HT group.

### Evaluation of left atrial functions using 2D-STE

Phasic strain values of the left atrium (LASr, LAScd, and LASct) showed significant differences among the groups (*p* < 0.001). In the non-dipper hypertension group, LASr (27.1 ± 3.3), LAScd (15.1 ± 2.7), and LASct (12.2 ± 1.9) values were significantly lower compared to the control and dipper hypertension groups (*p* < 0.001). The control group had the highest strain values (LASr: 42.4 ± 6.8, LAScd: 22.5 ± 8.5, LASct: 19.8 ± 5.1, Table 4).

**Table 4:** Left atrial strain analysis with Speckle Tracking Echocardiography of study patients.

Variables	Control group (n = 35)	Dipper HT group (n = 50)	Non-dipper HT group (n = 50)	*p*
LASr	42.4 ± 6.8^a,c^	34.4 ± 3.9^b,c^	27.1 ± 3.3^a,b^	<0.001
LAScd	22.5 ± 8.5^a,c^	18.5 ± 3.7^b,c^	15.1 ± 2.7^a,b^	<0.001
LASct	19.8 ± 5.1^a,c^	15.6 ± 1.7^b,c^	12.2 ± 1.9^a,b^	<0.001

*HT* hypertension, *LA-ɛsys* left atrial reservoir strain, *LA-ɛe* left atrial conduit strain (Early Diastole), *LA-ɛa* left atrial pumping strain (Late Diastole).

ANOVA test was used for continuous variables with post-Hoc Bonferroni correction. Categorical variables were compared by the x2 test. For post-hoc analysis results are shown as;

^a^Significantly different compared to the Dipper HT group.

^b^Significantly different compared to the Control group.

^c^Significantly different compared to the Non-dipper HT group.

### Role of echocardiographic left atrial measurements in assessing associations with hypertensive nephropathy

LASr was determined as the only significant variable associated with hypertensive nephropathy. Logistic regression analysis revealed a negative relationship between LASr and hypertensive nephropathy (β = −1.099, *p* < 0.001, Table 5).

**Table 5:** Univariate and multivariate logistic regression analysis of predictors for hypertensive nephropathy.

Variables	B	*P* value	95% Confidence Interval		β*	*P* value	95% Confidence Interval	
			Lower	Upper			Lower	Upper
LASr	−0.479	0.040	0.392	0.977	−1.099	<0.001	0.202	0.551
LAScd	0.468	0.064	0.974	2.619	-	-	-	-
LASct	0.246	0.279	0.819	1.997	-	-	-	-
LAVI maximum	−0.25	0.437	0.415	1.463	-	-	-	-
LAVI minimum	0.209	0.487	0.683	2.225	-	-	-	-
LA diameter	0.012	0.921	0.798	1.283	-	-	-	-
IVS thickness	−0.049	0.955	0.174	5.212	-	-	-	-
Posterior Wall thickness	0.277	0.755	0.232	7.518	-	-	-	-
LVMI	0.010	0.355	0.989	1.030	-	-	-	-

*LAVI* left atrial volume index, *LVMI* left ventricular mass index, *IVS* interventricular septal, *LASr* left atrial reservoir strain, *LAScd* left atrial conduit strain (Early Diastole), *LASct* left atrial pumping strain (Late Diastole).

Logistic regression analysis was performed to evaluate predictors of hypertensive nephropathy. Significant predictors included LASr, indicating a strong negative association. Confidence intervals and *p*-values represent statistical significance at the 95% level. B: unstandardised regression coefficient, β: standardised regression coefficient, *: F:2.347, R2:0.230, *p* < 0.001.

### Role of echocardiographic left atrial measurements in assessing associations with hypertensive heart disease

LA diameter, LASr, and LAVI maximum values were identified as significant variables associated with hypertensive heart disease. Logistic regression analysis demonstrated a positive association between LA diameter (β = 0.086, *p* < 0.001) and LAVI maximum (β = 0.097, *p* = 0.003) with hypertensive heart disease. In contrast, a negative association was observed between LASr and hypertensive heart disease (β = −0.134, *p* < 0.001, Table 6).

**Table 6:** Univariate and multivariate logistic regression analysis of predictors for hypertensive heart disease.

Variables	B	*P* value	95% Confidence Interval		β*	*P* value	95% Confidence Interval	
			Lower	Upper			Lower	Upper
LASr	−0.484	0.032	0.412	0.934	−0.134	<0.001	0.815	0.925
LAScd	0.152	0.215	0.804	1.459	-	-	-	-
LASct	0.087	0.478	0.784	1.345	-	-	-	-
LAVI maximum	0.316	0.045	1.008	1.892	0.097	0.003	1.045	1.123
LAVI minimum	0.056	0.674	0.912	1.691	-	-	-	-
LA diameter	0.145	0.038	1.012	1.289	0.086	<0.001	1.041	1.126

*LAVI* left atrial volume index, *LASr* left atrial reservoir strain, *LAScd* left atrial conduit strain (Early Diastole), *LASct* left atrial pumping strain (Late Diastole).

Logistic regression analysis was performed to evaluate predictors of hypertensive heart disease. Significant predictors included LASr, indicating a strong negative association, and LAVI max and LA diameter, both showing positive associations. Confidence intervals and *p*-values represent statistical significance at the 95% level.Confidence intervals and *p*-values represent statistical significance at the 95% level. B: unstandardised regression coefficient, β: standardised regression coefficient, *: F:2.347, R2:0.230, *p* < 0.001.

## Discussion

In our study, the results showed that the non-dipper hypertension group exhibited significantly higher rates of hypertensive heart disease and nephropathy compared to the dipper hypertension and control groups. Additionally, this group demonstrated significantly lower left atrial strain values, further reflecting impaired atrial mechanics. Left atrial parameters, especially LASr, which was significantly associated with both hypertensive heart disease and hypertensive nephropathy, were identified as strongly associated with target organ damage.

### Clinical significance of hypertension and end-organ damage

Hypertension remains a significant global health concern, contributing substantially to morbidity and mortality while imposing a considerable burden on healthcare systems in both developed and developing countries [27]. Non-dipper hypertension, characterized by inadequate nocturnal blood pressure reduction, is associated with a heightened risk of end-organ damage and worsened clinical outcomes [28, 29]. In our study, the higher prevalence of hypertensive heart disease in the non-dipper hypertension group underscores the substantial chronic pressure load and hemodynamic stress on the heart in this patient population. Similarly, the markedly increased prevalence of hypertensive nephropathy in non-dipper hypertension highlights the necessity of vigilant renal function monitoring in these patients. Multiple studies have established a strong association between non-dipper hypertension and end-organ damage, emphasizing the accelerated progression of irreversible injury in target organs when nocturnal blood pressure remains uncontrolled [29, 30]. Kario et al. [31] demonstrated that elevated nocturnal systolic blood pressure load is linked to myocardial fibrosis, renal microvascular injury, and an increased risk of stroke [31]. These findings underscore the importance of targeting nocturnal blood pressure control as a critical therapeutic goal in this subgroup. These findings highlight the need for specific biomarkers, such as left atrial parameters, to better understand the cardiovascular and renal consequences of non-dipper hypertension.

### Impact of hypertension on left atrial volume and physiomechanics

Hypertension exerts its deleterious effects on the cardiovascular system not only through elevated arterial pressure but also via hemodynamic changes affecting cardiac chambers, particularly the left atrium [32, 33]. Our study demonstrates the adverse effects of hypertension on left atrial volume and physiomechanics, supporting this evidence. While Nagueh et al. highlighted the utility of left atrial parameters in detecting diastolic dysfunction, our study demonstrates their association with both cardiac and renal dysfunction [26]. These findings underscore the importance of assessing left atrial functions not only as indicators of cardiac dysfunction but also as potential biomarkers for hypertension management.

### Association of left atrial parameters for end-organ damage

In the existing literature, left atrial strain values are recognized as valuable parameters for assessing associations with cardiovascular diseases [25, 34, 35]. Our study supports the potential role of these parameters in hypertensive disease. According to our results, LASr has been identified as significantly associated with both hypertensive heart disease and hypertensive nephropathy. The inverse relationship between LASr and microalbuminuria may reflect early renal microvascular injury in hypertensive patients. Moreover, LASr may serve as a significant marker of hypertensive heart disease. These findings support the role of left atrial phasic parameters in guiding renal protection strategies and optimizing hypertension management.

### Clinical implications and future directions

The findings of this study underscore the multifaceted impact of non-dipper hypertension on target organ damage, particularly the heart and kidneys. The strong association of left atrial parameters with target organ damage highlights their potential as noninvasive biomarkers for assessing risk and aiding in clinical decision-making. Moving forward, incorporating advanced echocardiographic parameters into routine hypertension evaluation could facilitate tailored therapeutic interventions, particularly in high-risk subgroups such as non-dipper hypertensive patients. Longitudinal studies focusing on left atrial parameters could validate their role in guiding personalized therapeutic strategies, particularly in mitigating the risk of progressive organ damage in high-risk hypertensive subgroups. Additionally, interventional studies evaluating the impact of targeted nocturnal blood pressure reduction strategies on left atrial function and end-organ outcomes would provide critical insights into optimizing hypertension management.

### Limitations of the study

This study provides valuable insights into the impact of hypertension on left atrial function and end-organ damage; however, it has several limitations. First, the single-center design and relatively small sample size may limit the generalizability of the findings to broader populations. Second, the lack of long-term follow-up precluded the evaluation of the effects of hypertension on left atrial remodeling.

Given these limitations, future research should focus on follow-up studies that can support the clinical relevance of the findings.

## Conclusion

This study highlights the significant impact of hypertension, particularly in the non-dipper subgroup, on left atrial physiomechanics and end-organ damage. Left atrial strain measurements have emerged as robust biomarkers for assessing risk for complications such as hypertensive heart disease and nephropathy. Our findings suggest that integrating left atrial parameters into routine clinical assessments for hypertension management holds substantial potential to improve patient outcomes.

Future studies with larger sample sizes and long-term follow-up data are needed to validate these results and further investigate the role of left atrial parameters in guiding personalized treatment strategies for hypertensive patients.

## Summary

### What is known about the topic:

- Non-dipper hypertension is a phenotype associated with an increased risk of target organ damage.
- Left atrial remodeling is an early indicator of cardiac dysfunction related to hypertension.
- Speckle-tracking echocardiography is a reliable method for assessing left atrial strain.

### What this study adds:

- Significant impairment in left atrial strain values was observed in the non-dipper hypertension group, which also demonstrated higher rates of target organ damage (hypertensive heart disease and nephropathy).
- LASr (reservoir strain) has been identified as strongly associated with hypertension-related target organ damage (cardiac and renal).
- This study strongly supports the use of LASr as an effective, cost-efficient, and non-invasive biomarker for assessing associations with target organ damage in clinical practice.

## Data Availability

The data that support the findings of this study are available from the corresponding author upon reasonable request.
